# Identifying barriers to ART initiation and adherence: An exploratory qualitative study on PMTCT in Zambia

**DOI:** 10.1371/journal.pone.0262392

**Published:** 2022-01-13

**Authors:** Tukiya Kanguya, Aybüke Koyuncu, Anjali Sharma, Thankian Kusanathan, Martha Mubanga, Benjamin H. Chi, Michael J. Vinikoor, Mwangelwa Mubiana-Mbewe

**Affiliations:** 1 Centre for Infectious Diseases Research in Zambia, Lusaka, Zambia; 2 Department of Gender Studies, University of Zambia, Lusaka, Zambia; 3 University of North Carolina at Chapel Hill, Chapel Hill, North Carolina, United States of America; 4 University of Alabama at Birmingham, Birmingham, Alabama, United States of America; Centre for Sexual Health & HIV/AIDS Research (CeSHHAR)R, ZIMBABWE

## Abstract

**Background:**

Though antiretroviral therapy (ART) is widely available, HIV positive pregnant women in Zambia are less likely to start and remain on therapy throughout pregnancy and after delivery. This study sought to understand readiness to start ART among HIV pregnant women from the perspectives of both women and men in order to suggest more holistic programs to support women to continue life-long ART after delivery.

**Methods:**

We conducted a qualitative study with HIV positive pregnant women before and after ART initiation, and men with female partners, to understand readiness to start lifelong ART. We conducted 28 in-depth interviews among women and 2 focus group discussions among male partners. Data were transcribed verbatim and analyzed in NVivo 12 using thematic analysis. Emerging themes from the data were organized using the social ecological framework.

**Results:**

Men thought of their female partners as young and needing their supervision to initiate and stay on ART. Women agreed that disclosure and partner support were necessary preconditions to ART initiation and adherence and, expressed fear of divorce as a prominent barrier to disclosure. Maternal love and desire to look after one’s children instilled a sense of responsibility among women which motivated them to overcome individual, interpersonal and health system level barriers to initiation and adherence. Women preferred adherence strategies that were discrete, the effectiveness of which, depended on women’s intrinsic motivation.

**Conclusion:**

The results support current policies in Zambia to encourage male engagement in ART care. To appeal to male partners, messaging on ART should be centered on emphasizing the importance of male involvement to ensure women remain engaged in ART care. Programs aimed at supporting postpartum ART adherence should design messages that appeal to both men’s role in couples’ joint decision-making and women’s maternal love as motivators for adherence.

## Introduction

In Zambia, HIV infection due to mother-to-child transmission (MTCT) is 706 per 100,000 births [1]. To eliminate new pediatric HIV infections by 2021, the Zambian Ministry of Health (MOH) adopted the Elimination of Mother-to-Child Transmission of HIV (EMTCT) initiative. The last mile towards EMTCT requires >90% ART coverage at country level by 2021 and that 95% of HIV positive pregnant women be on treatment and are retained in care [1]. Data from the Southern African region have shown that women who initiated ART during pregnancy had a poor 24-month retention in care, and this contributed to an increasing number of children being infected during the breastfeeding period [2]. Approximately 60% of new HIV infections in children occur during the breastfeeding period due to poor maternal antiretroviral therapy (ART) adherence and inadequate systems of follow-up for postpartum mothers [3]. Therefore, supporting pregnant women to identify and overcome barriers to long-term ART use is a priority to eliminate MTCT in the country. Despite these efforts made by MOH, both ART initiation and adherence among HIV infected pregnant women remain barriers to reaching this goal by 2021.

Barriers to timely ART initiation are diverse, however women in sub-Saharan Africa consistently report the need to discuss their HIV status with their husbands before deciding to start ART [4–6]. A major deterrent to ART initiation and sustained adherence among pregnant women infected with HIV is the lack of male involvement in ART care [7,8]. Men’s attitudes towards joining Prevention of Mother-to-Child Transmission (PMTCT) in sub-Saharan Africa have been linked to cultural expectations and the social norms that hold women responsible for attending PMTCT [8]. Men in Botswana regarded antenatal care (ANC) facilities as being "generally unfriendly" to them [9]. Most recently, a review of studies incorporating men suggested that male "support" as well as "involvement" are key to increasing PMTCT uptake [10]. The WHO 2010–2015 guidelines on PMTCT emphasize the need to involve male partners to scale up PMTCT services in sub-Saharan Africa [11]. Despite this, there is limited literature on the perspectives of male partners on their involvement in ART care and why women delay ART initiation or disengage from care after delivery.

Achieving the EMTCT goal to eliminate new pediatric HIV infections by 2021 will require a deeper understanding of the barriers towards both initiation and retention on ART among pregnant women infected with HIV from the perspective of both men and women. To address this knowledge gap, we conducted a formative qualitative research study to better understand readiness to start lifelong ART among pregnant women infected with HIV.

## Methods

### Research setting

This study was conducted in two urban health centers in Lusaka District, Zambia, approximately one year after introduction of Option B+ in Zambia (i.e., universal and lifelong ART for all pregnant and breastfeeding women living with HIV). Health centers were purposively sampled in consultation with the Lusaka District Health office to include urban government facilities with medium to high patient volume and physical space for study activities at the clinic. Lusaka district is one of four districts within Lusaka Province, which is home to approximately 20% of Zambia’s population of 17 million [12]. Most residents in Lusaka District live below the poverty line in high-density peri-urban slums or “compounds” with poor access to safe water and sanitation. Similar to many countries in sub-Saharan Africa, the HIV epidemic disproportionately impacts individuals living in urban areas in Zambia with Lusaka province having the highest prevalence of HIV infection among adults (15.7%) [13].

### Study procedures

The objective of this study was to understand readiness to start lifelong ART among HIV-infected pregnant women. Findings from this study were subsequently used to develop and evaluate a quantitative tool to assess ‘readiness’ for ART initiation and an intervention package aimed at supporting adherence and retention on treatment among the pregnant population, for which results are presented elsewhere [14].

This project was reviewed in accordance with Centers for Disease Control and Prevention (CDC) human research protection procedures and was determined to be research, but CDC investigators did not interact with human subjects or have access to identifiable data or specimens for research purposes. Ethical approval for the study was obtained from the University of Zambia Biomedical Research Ethics Committee (# 015-11-13) and the University of North Carolina at Chapel Hill Institutional Review Board (# 13–3884).

### Participants

HIV-infected pregnant women not yet on ART (pre-ART group) and HIV-infected pregnant or postnatal (≤42 days after delivery) women on ART (post-ART group) were recruited to participate in in-depth interviews (IDIs). Partners of women who were recently or currently pregnant were also recruited into the study to participate in focus group discussions (FGDs) in an effort to understand male perspectives on barriers and facilitators of ART initiation and adherence. Male partners may or may not have been the partners of women participating in IDIs, and did not necessarily have partners living with HIV. All participants in the various study groups were ≥18 years old, did not have a known history of mental illness, were able to communicate in one of the three languages used in the study (English, Nyanja or Bemba) and provided written informed consent to participate in the study. The target sample size needed to reach saturation of themes and patterns emerging from data related to the beliefs, behaviors, and experiences of men and women in each study group was determined prior to sampling and data collection [15,16].

Study participants were recruited using convenience sampling. Women presenting at ANC visits or under-5 clinics at the selected urban health centers were sensitized about the study during the daily routine group health talks. Women interested in the study were screened for study participation. In addition, women were identified through their health records, in collaboration with the health facility staff, and all those meeting inclusion criteria were approached on an individual basis for study participation. All eligible women were recruited after providing informed consent. Sensitization about the study for men was done in the general outpatient clinic, TB or ART clinics at the same health facilities. Men were approached individually to establish interest and those interested were screened for study participation. In addition, community sensitization was done through community leaders; men were then approached individually in the community in various places including markets, bus-stops, churches and other public spaces. Those interested in participating were then invited to the study site and screened for study participation. Eligible men were recruited after providing informed consent. Recruitment of men outside the health facilities was done in order to minimize bias towards men with positive health-seeking behavior.

### Data collection

Data were collected between June and September 2015. Topics shown previously to be important when measuring ART readiness were covered in IDIs, namely: disclosure, partner involvement, psychosocial issues, HIV medication beliefs, and alcohol and drug use [17]. To understand individual barriers and facilitators of ART initiation, we assessed women’s knowledge and understanding of ART, their experiences with stigma and discrimination, their existing support structures, and their individual motivations (see S1–S3 Files). HIV-infected pregnant women were enrolled and interviewed before or within seven days of ART initiation (pre-ART group) and then a subset of these women was asked to participate in an additional IDI 2–3 months after their first IDI to assess changes in barriers to initiation over time. Participants who had already initiated ART at presentation (post-ART group) were asked about barriers and facilitators to ART adherence, with topics including: social support, distance from clinics and transportation, cultural norms, partner involvement, patient-provider relationships, HIV-related stigma, and experiences at health facilities. Participants were asked to assess the quality and effectiveness of adherence services already received (e.g., adherence counselling); they were also asked for suggestions for additional or alternative services that could be provided to help improve adherence to ART.

FGDs with men who currently have female partners who are/were recently pregnant concentrated on their knowledge about HIV and PMTCT, factors that impact starting and adhering to ARVs among pregnant women, and the level of men’s engagement in their partner’s healthcare during and after pregnancy defined as support in the home or at clinic visits.

All IDIs and FGDs were conducted in English, one of Zambia’s official language, or one of two local languages (Nyanja and Bemba) by research staff fluent in all three languages, and were audio-recorded. IDIs and FGDs were held in the language of participant preference, which was determined at the beginning of each FGD, and held in a private setting at one of the two study clinics or the Centre for Infectious Disease Research in Zambia (CIDRZ) research facility in Lusaka, Zambia.

### Analysis

Audio recordings of IDIs and FGDs were transcribed verbatim and translated from local languages of Nyanja and Bemba to English by trained research assistants, where necessary. The social ecological framework [18] was selected *a* priori to organize emerging themes for barriers and facilitators of ART initiation and adherence. The social ecological model is a theory-based framework for understanding the interactive effects of individual, interpersonal, health system level and structural factors of behavior. We defined individual level factors as those within a woman’s control and awareness; interpersonal level factors as a woman’s primary relationships affecting her ART treatment, health system level factors as health care structure and design; and structural level factors as women’s socio-economic environment.

Within levels of the social ecological model, two coders used inductive thematic analysis to code the text in the data. Inductive thematic analysis is a comprehensive process involving reading through the transcripts for familiarization and identifying emerging key themes and codes which are then entered into a codebook [19]. Coding was compared amongst the two coders for consistency and similarity. The categorization and labeling of emergent themes were reviewed, defined and standardized by the two coders (AK, TK) and any discrepancies in coding were resolved by an independent qualitative expert (AS). Once the emergent themes were reviewed, these were defined and appropriately labeled by the two coders. All data were coded using NVivo 12 (QSR International, Melbourne, Australia) software.

## Results

### Characteristics of study population

We conducted 28 IDIs among 20 pregnant and postpartum HIV-infected mothers and 2 FGDs among 16 men in groups of 8. Among 20 women, 12 had IDIs at only one time-point (5 in pre-ART group, and 7 in post-ART group) and 8 pre-ART women had a follow-up IDI that took place 2–3 months after their first interview. Of the 28 IDIs, 5 were conducted in English while 23 were conducted in local languages and later translated into English (n = 2 IDIs in Bemba; n = 21 IDIs in Nyanja). FGDs with men used a mix of the 3 languages. Demographic characteristics of the women and men are shown in Tables 1 and 2 respectively. IDIs that occurred only at one time-point were conducted among a mix of pregnant (n = 7) and postnatal (≤42 days after delivery) (n = 5) women.

**Table 1:** Characteristics of pregnant and postnatal HIV-infected women participating in in-depth interviews (IDIs), Lusaka, Zambia, 2015.

Characteristic	N (%) or Median (IQR)
**Total = 20**	
Age, years	31 (29–37)
Number of children before the current pregnancy	2 (2–4)
Marital status	
Cohabiting/Married	18 (90.0)
Missing	2 (10.0)
Educational level	
Primary	4 (20.0)
Secondary	14 (70.0)
Tertiary	1 (5.0)
Missing	1 (5.0)
Primary occupation	
Not employed or keeps household	11 (55.0)
Formally employed ^A^	2 (10.0)
Businesswoman or self employed	6 (30.0)
Missing	1 (5.0)
Time since HIV diagnosis	
< 1 month	5 (25.0)
1–12 months	6 (30.0)
13–24 months	3 (15.0)
>24 months	2 (10.0)
Missing	4 (20.0)
Initiated ART at/before IDI	17 (85.0)
Clinic	
Facility 1	9 (45.0)
Facility 2	11 (55.0)

^A^ Employed in an organization, company, institution, or shop.

ART = antiretroviral therapy.

**Table 2:** Characteristics of male partners participating in the FGDs in Lusaka, Zambia, 2015.

Characteristic	N (%) or Median (IQR)
**Total = 16**	
Age, years	38 (28–45)
Marital status	
Cohabiting/Married	16 (100.0)
Primary occupation	
Not employed or keeps household	2 (12.5)
Formally employed ^A^	6 (37.5)
Businessman or self employed	6 (37.5)
Occasionally Employed	1 (6.3)
Informally Employed	1 (6.3)
Highest Level of Education	
Primary	1 (6)
Secondary	10 (63)
Tertiary	5 (31)
Health Facility ^B^	
Facility 1	8 (50)
Facility 2	8 (50)

^A^ Employed in an organization, company, institution, or shop.

^B^ Health facility and corresponding catchment area where recruitment took place.

Because immediate ART initiation had become standard of care, at the time of their first IDI, 17/20 mothers (85.0%) had initiated on ART and 1 mother (4.8%) indicated plans to initiate the day of her interview (Table 1). Only 1 participant who had not initiated ART at the time of their first IDI had a subsequent follow-up IDI, and initiated ART prior to her follow-up interview. All participants with follow-up interviews who had initiated ART at the time of their first IDI (n = 7) reported still being on ART at the time of their follow-up interview.

Both women and men were very knowledgeable about HIV care in pregnant women and PMTCT. This knowledge included understanding ANC visits, recommended diet while on ART and type of medication that women are supposed to take.

### Barriers and facilitators to ART initiation

Pregnant and postnatal women and men in this study reported a range of barriers to ART initiation within all hierarchies of the social-ecological model (individual, interpersonal, health system, and structural levels). However, both men and women agreed that women found it most difficult to overcome individual and interpersonal barriers to ART initiation. Both women who had already initiated and not yet initiated ART viewed partner disclosure and support as necessary for ART initiation. A participant who had not initiated ART at the time of her interview explained:

“*I always think of how I am I going to manage taking ARVs without my partner knowing*, *because definitely he has to know about me being on treatment*. *Since I have never disclosed my status*, *I feel it is unfair for me to start taking ARVs without my partner knowing*.*” (Female*, *31 years*, *had not initiated ART)*.

Barriers to partner disclosure, such as fear of partner blame or divorce, were reported as substantive barriers to ART initiation among both pregnant and postnatal women:

“… *It’s not like I tested HIV positive today*, *it has been two years*. *[I have] not disclosed my status to him because there are a lot of fears*. *He might end up leaving me*… *I think the main challenge that women have is actually disclosure*. *If I go to a clinic and I am tested alone and am HIV positive*, *what if he asks me what made me do it*? *Maybe he would say that*, *‘You know what you have been doing*, *that is why you went for a test’; or he may end up accusing me of having extramarital affairs*, *that is why I went quiet*… *It is very difficult if am tested alone for me to go and tell my partner that I tested positive*.*” (Female*, *31 years*, *had not initiated ART)*.

Similar to the women’s perspectives, men thought that fear of divorce or spousal separation impeded ART initiation among married women. Both women and men highly valued and guarded their marital relationship. Having a healthy and positive relationship with their spouse superseded individual physical health. Men repeatedly reported that women were afraid of the marital repercussions of telling them their status and as a result would not take ARVs:

“*They fear being divorced and also fear the rejection by families*.*” (Male*, *39 years)*

“*Another thing is the fear of being divorced*. *They may say that if I have not disclosed to my husband and start taking ARVs without him knowing*, *the marriage will come to an end*. *So mostly they want to protect their marriage*, *they do not want to be divorced and this is why they do not feel ready to start taking their drugs*.*” (Male*, *29 years)*

Only 2 participants indicated that they did not have plans to initiate ART at the time of their interview. Both had not disclosed their status to their partner, and suggested couples testing and counselling as strategies to support partner disclosure. Suggestions for the need for couples testing and counselling were further strengthened by descriptions from delayed ART acceptors (those who did not accept ART the day they tested HIV positive) of the need to go home and discuss with their partner prior to initiating ART:

“*You know when you have just been told news like you are HIV positive*, *it is always hard to accept*. *So*, *I had to sit and discuss with my partner; so time passed before we made a decision to start taking ARVs*. *We finally accepted our status*, *my partner and I then agreed that I start taking ARVs just yesterday*.*” (Female*, *32 years*, *on ART)*.

#### Discordant relationships

HIV negative men in discordant relationships reported that their female partners suffered emotional distress. Women in discordant relationships viewed themselves as being marginalized and struggled with understanding how they acquired HIV despite being faithful; these feelings delayed their acceptance of ART. One man thought that women may feel more emotionally supported if their partner was HIV positive:

“*On the other hand*, *I still feel psychologically that she would have preferred it if both of us were HIV positive and taking drugs because she feels marginalized*. *[Our sero-discordant status] is something that even I have failed to understand*, *it’s just God’s doing*. *I have no words to ask why and why not because God himself knows better*.*” (Male*, *22 years)*

#### Preference for antenatal HIV testing

At the time of the study, the available HIV testing options were: routine provider-initiated testing (STI, antenatal and TB clinics), Voluntary Counselling and Testing, Community Based (door-to-door, mobile outreach) and diagnostic testing. Men largely felt that women preferred testing for HIV in antenatal rather than ART clinics because they thought of them as more convenient and comfortable. Men reported that their partners tested for HIV at antenatal visits, preventing them from HIV testing as a couple. Because the men were initially unaware of their partner’s status, it was difficult for them to be involved in the health care of partner and also to provide support.

“*They don’t want to have their HIV test done at voluntary counselling and testing (VCT) so that they know if they are positive or negative but they prefer it done at antenatal*. *It’s like that’s where they feel comfortable*.*” (Male*, *39 years)*

“*Women don’t want to go for VCT so the best thing to do is to accompany them*.*’’ (Male*, *29 years)*

#### Maternal love

In instances of disclosure and partner support, wanting to protect the health of the baby and love for one’s children were facilitators to acceptance of one’s HIV status and readiness to initiate ART. While women described wanting to be healthy and prolong own life as other important individual-level motivators for ART initiation, for many participants these individual desires had substantial interactive effects with maternal love. A mother’s love for her unborn child was sufficient to overcome not only individual barriers such as lack of acceptance of one’s status, feeling healthy, lack of confidence in ability to follow ARV regimens, and fear of harm/side-effects due to ARVs, but also interpersonal and community level barriers such as fear of stigma and health system barriers such as long queues at clinics. Maternal desires to protect their children uniquely instilled a self-responsibility to overcome other existing barriers to ART initiation:

"*You find people who say bad things about HIV positive people who take ARVs*. *So*, *it is up to you to ignore all those bad things people say and continue taking ARVs to help yourself*. *If you take your ARVs you will live long enough to keep your children*, *do not listen to what people say*.*" (Female*, *29 years*, *on ART)*.

### Barriers and facilitators to ART adherence

All women demonstrated proficiency and understanding regarding how ARVs function within the body and the importance of taking doses at the right time. Most women who had initiated ART at the time of the interview expressed no difficulties taking ARV regimens as prescribed by health care workers and only reported few instances of missing doses. Self-reported adherence, however, was not attributable to a lack of barriers to ART adherence by the women but rather a self-responsibility to overcome these barriers.

Both women and men described barriers to adherence in all hierarchies of the social-ecological framework. Reported barriers included a lack of partner disclosure, rumors and misinformation about HIV and ARVs spread by faith healers, difficulties getting permission from work to pick up ARVs, and long queues at the clinic. Despite these barriers, an emergent theme was women’s intrinsic motivation to “be serious” with their ARVs and overcome barriers to adherence. For example, when asked why long queues at a clinic could be a barrier to adherence, one participant explained:

“*That person may not just be serious about their life because mostly when people who are not serious come to the clinic for appointments and find that there are a lot of people in the queue they will go back*. *Mostly such people are drunkards and not just serious about their lives*, *that is what causes them to stop*. *But if you really want to have a good and healthy life*, *you can still wait and get your medication*.*” (Female*, *23 years*, *on ART)*

Women had diverse opinions on adherence support strategies suggested by the interviewer such as peer educators, SMS reminders, adherence counselling, and material incentives. Some women believed the strategies could be helpful and others believed the strategies would be ineffective or could unintentionally disclose one’s status to partners or community members (SMS reminders and follow-up by peer educators). The opinion that strategies would be ineffective was based on the belief that adherence had to be intrinsically motivated as explained by one participant:

“*It [incentivizing] may look as if someone is begging you to live when this is about your life and you just need to encourage yourself*.*” (Female*, *unknown age*, *had not initiated ART)*.

Similar to ART initiation, partner support, and the desire to continue taking care of one’s children and watching them grow, were seen as significant motivators for adherence to ART. Strategies suggested by participants to support adherence included partner and family reminders, putting ARVs somewhere in the home where one can always see them, and always carrying drugs on one’s person. Men believed they were the first source of support in helping improve adherence, followed by children in the household.

“*The strategies—firstly it is disclosure*, *upon disclosing to your husband or children*, *they need know what time you will be taking the drugs so that they can remind you in case you forget” (Male*, *21 years)*

“*There are also psychological reminders like if you have a daughter*, *you may not disclose to her the status of her mother but just tell her that your mother will be taking BP [high blood pressure] medication and you should be reminding her every day*. *You even tell her that this is the responsibility that I have given you and maybe even give her a certain amount of money for lunch at school*. *That is just a psychological way but wherever she is she will be reminded that she has to remind her mother to take her medication*.*” (Male*, *22 years)*

When asked which one strategy would be best for supporting adherence, women reported setting alarms on one’s phone or watch as the best strategy for ART adherence. Alarms were described by women as an effective strategy for supporting adherence that did not jeopardize confidentiality of one’s HIV status and could thus be utilized both by individuals who had and had not disclosed their HIV status to their partners and peers:

“*The alarm is the most important thing and if you are scared that people will ask when it rings*, *you can put it on vibration*. *The phone is the most effective way for remembering to take ARVs*.*” (Female*, *23 years*, *on ART)*.

Men similarly perceived alarm reminders as helpful in improving adherence among pregnant women. The men reported that they would set alarms on their phones to remind their partners to take medication. Having a fixed time set for taking medication as a couple improved ART uptake among the women:

“*On the other hand you have to put a reminder on the phone; an alarm which when it rings*, *reminds you to take your drugs*.*” (Male*, *21 years)*

In addition to alarm reminders, according to the men, women needed to be guided by them and take ART under supervision; saying that, “men were the lions and women the sheep”. They reported that men led women to start and remain on ARVs because women went through a phase of denial about their HIV diagnosis.

“*Women also behave like children*. *If you give them medicine*, *some hide them under the pillow or even throw them away and sometimes they complain that from the time they started taking the same medicine they have never seen any change*. *So monitoring is required*.*” (Male*, *32 years)*

### Social ecological framework on barriers and facilitators to ART initiation and adherence

**Fig 1:**
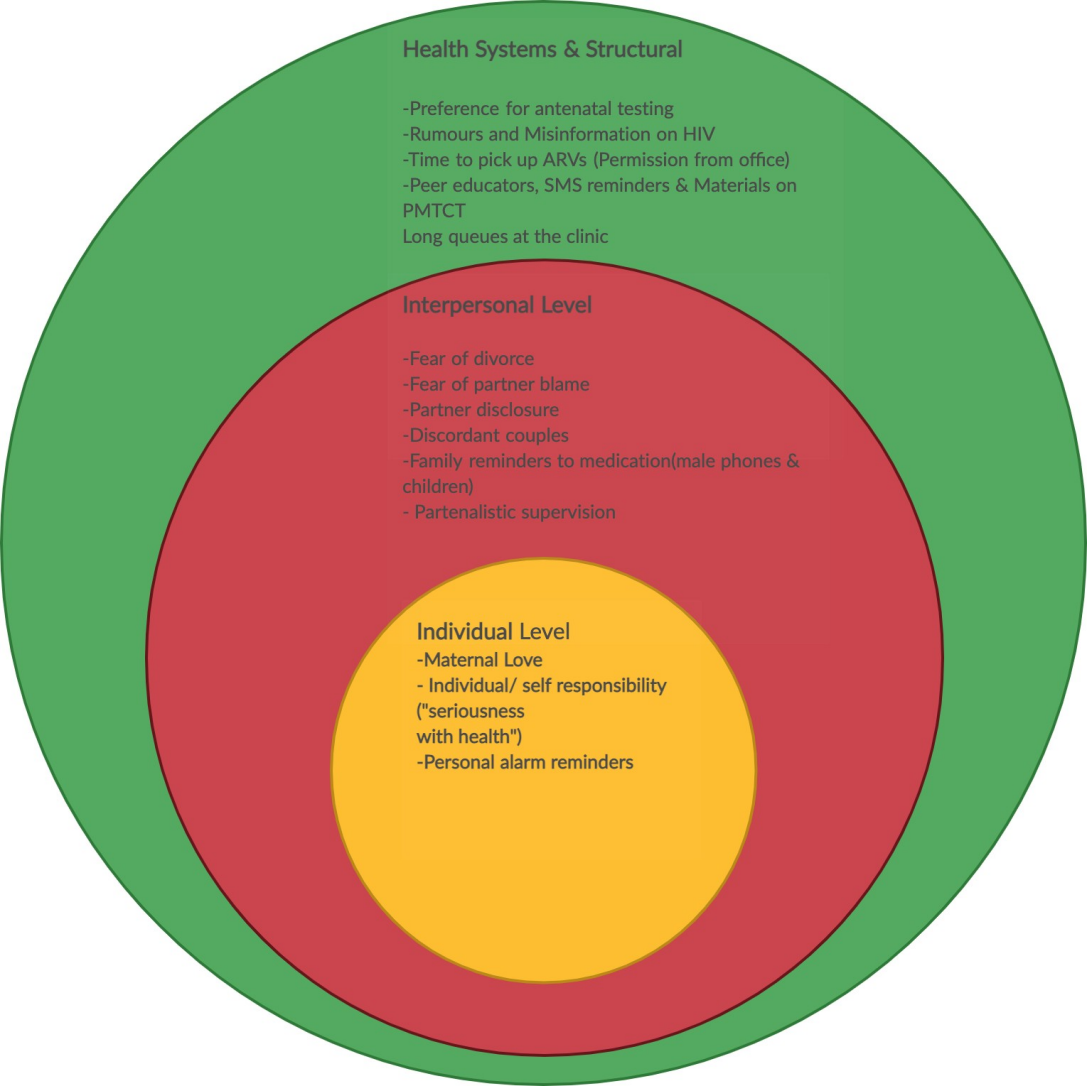
Social ecological framework on barriers & facilitators to initiation and adherence among women and men. Source: Adapted and developed by the authors.

Barriers and facilitators to ART initiation and adherence identified in this study are summarized in Fig 1.

## Discussion

Our study identified partner disclosure as a critical facilitator for maternal ART initiation among Lusaka participants. The presence of partner disclosure and support at the interpersonal level was seen as sufficient to overcome all other self-reported socio-ecological barriers to ART initiation. While both women and men identified male partners as facilitators of ART adherence, they valued different types of support. Men emphasized constant supervision and leadership to ensure women adhere to ART. Women, on the other hand, emphasized the need for open and non-punitive interactions. These results add to research in other low and middle-income countries (LMIC) offering a more nuanced understanding of partner support to help guide appropriate messaging to increase uptake of Option B+ [7,20–27].

A lack of male knowledge of HIV and PMTCT has previously been identified in numerous studies in sub-Saharan Africa as a barrier to male involvement in PMTCT, partner disclosure, and women’s ART initiation and adherence [7,20–22,27]. The relatively high knowledge levels on HIV, PMTCT, and benefits of ARVs among men in our study simultaneously underscore reach of PMTCT promotion but also the inadequacy of the messaging in tailoring male involvement to the expressed needs of their HIV positive pregnant and breastfeeding partners. Our results suggest that interventions aimed at addressing barriers to disclosure, initiation, and adherence could benefit from appealing to the value both men and women place on their relationship as a strategy to increase male involvement in joint decision-making for women’s HIV care.

Reaching the last 5–10% of women in PMTCT elimination goals will require creative strategies. For example, interventions such as HIV self-testing have shown the potential to improve disclosure among couples. In Malawi, a randomized control trial on HIV self-testing was successful in promoting couples testing [28]. Our findings utilize data from 2015, therefore barriers and facilitators for ART initiation and adherence among pregnant and postpartum women may have subsequently changed. However, interventions targeting the barriers identified in this analysis have not been widespread, while low or sometimes unhelpful male involvement continues to be documented [29]. Innovative methods focused on the recruitment of men as pioneers in women’s ART initiation and adherence may still be a relevant and necessary strategy for addressing fear of divorce and partner blame as barriers among women who have not yet initiated ART, decreasing delayed acceptance of ART, and supporting ART adherence among pregnant and postpartum women [30].

Our results complement existing literature identifying partner disclosure and support as consistently important facilitators of ART initiation among pregnant women [7,20–27], but are to our knowledge, the first to suggest disclosure/support are both necessary and sufficient to overcome all other self-reported barriers to initiation when coupled with a mother’s love for her children. While participants in our study acknowledged similar barriers to ART initiation identified by existing research in sub-Saharan Africa such as community stigma and feeling healthy [20,24,31,32], in the presence of partner support, participants reported both self-efficacy and responsibility to overcome these barriers mediated by their love for their children. The narrative of self-efficacy in overcoming barriers to ART initiation and adherence was also reflected in the selection of phone-based alarms by both women and men in our study as an effective method for supporting ART adherence.

Our study revealed that both men and women viewed the importance of male partners in the decision making on ART initiation and adherence but differed in reports on their level of involvement. Most of the men believed they should be consulted for guidance on ART adherence as the leaders of the family whereas women desired emotional rather than directive support from the men. The results complement existing literature from Southern Africa which reinforces the notion of men as decision makers in the family and women as caregivers in HIV treatment [33–35]. Studies on gender norms in HIV treatment have shown that men felt their masculinity was threatened and weakened when they lacked control over HIV care of the household [35]. Tailored community-based interventions have the potential to increase male involvement in PMTCT and thereby reduce stigma in the community. For instance, community-based educational messaging depicting the importance of men in HIV care has the potential to increase both knowledge and awareness on PMTCT among men and in the community [36,37]. Interventions to address these gender norms in HIV care should depict men as leaders in the health care of the family, who are also able to be empathetic and provide emotional support, to encourage discussions on HIV care among partners.

Contrary to previous research conducted among people living with HIV (PLHIV) in Zambia in 2010–11 [31], dissatisfaction with HIV counselling and misinformation about PMTCT/ARVs were not prominent barriers to ART initiation among women in our study population. These findings may be suggestive of improvements to community sensitization and HIV counselling efforts in Zambia. The scale up of HIV care services and ARV delivery in Zambia is further reflected by contrasting the lack of prominent structural level barriers to ART initiation by pregnant mothers in our study with the views of PLHIV in Lusaka in 2010 who reported fear of long-term availability of treatment as a significant barrier to ART initiation [31].

Our finding that love for one’s children and desire to have healthy HIV-free babies were the main motivators for ART initiation and adherence is consistent with existing research [20,21,24,26,27]. However, existing concerns that maternal love may have less influence on ART adherence if the baby is HIV-negative at birth and/or after the breastfeeding period [7] were not supported by our study. Our findings suggest that women may continue taking ART in order to live long healthy lives and to take care of their children and watch them grow.

This study is subject to several limitations. Our ability to draw conclusions regarding the strength of a mother’s love for her children as a long-term facilitator of ART adherence is limited given that women enrolled in our study were either currently pregnant or ≤42 days postpartum at the time of their IDI; however, most of the women already had other children at the time of the interview. We also did not collect data on whether mothers with multiple children were tested for HIV during previous pregnancies. Our findings may also have limited generalizability given that women could only be recruited for our study if already attending ANC/under-5 clinics and exhibiting positive health seeking behavior related to their child’s health. We did not systematically collect data on the location of male recruitment or how many of the men were living with HIV at the time of the study, both of which may have influenced the male perspectives expressed in our study. Disclosure status was self-reported by women in the study and was not systematically asked to all study participants. Our study’s reliance on self-reported adherence may also limit our ability to identify barriers to ART adherence due to social desirability bias and women’s unwillingness to admit poor adherence, which may have contributed to the low prevalence of self-reported poor adherence in our study. Findings from similar qualitative research among HIV-infected pregnant women in sub-Saharan Africa have identified heterogeneity in self-reported adherence, suggesting that challenges with ART adherence may still be identified in the context of self-report [24]. Despite this, prospective follow-up with HIV-infected mothers after the breastfeeding period with more rigorous measurement of ART adherence is needed to confirm the findings of this study. Additional research is also needed to confirm the findings of this analysis given that our results may have limited generalizability to rural areas though increasing migration of rural populations into urban cities in the last decade may increase the relevance of our findings to rural areas in Lusaka and sub-Saharan Africa [38,39].

## Conclusion

While women reported barriers to ART initiation and adherence at all four levels of the social-ecological framework, interventions at the health systems level and structural level may have limited capabilities to facilitate ART initiation and adherence if they do not also address interpersonal barriers related to partner disclosure and support. To realize Zambia’s ambitious pediatric HIV elimination goals, PMTCT programs need to strongly promote male engagement and utilize behavioral interventions that address the real and perceived negative consequences of HIV status disclosure. Designing messaging that appeals to men’s role in couples’ joint decision-making for household healthcare and to women’s maternal love may motivate women to continue on ART after delivery.

## Supporting information

S1 Checklist COREQ (COnsolidated criteria for REporting Qualitative research) checklist. (PDF)

S1 File Interview guide for women pre-ART. (PDF)

S2 File Interview guide for women post-ART. (PDF)

S3 File Interview cards. (PDF)
