# Supplementary material for: Identifying barriers to ART initiation and adherence: An exploratory qualitative study on PMTCT in Zambia
Source: PLoS One. 2022 Jan 13;17(1):e0262392. doi: 10.1371/journal.pone.0262392 (PMC8757984; doi:10.1371/journal.pone.0262392)
Supplement: S1 File — (PDF) [file pone.0262392.s002.pdf]

Participant ID number: ☐☐☐☐☐

Date: ☐☐/☐☐/☐☐☐☐

ART Readiness among Pregnant Women (Phase 1)
Interview Guide, Version 1.2, dated 13 April 2014
WOMEN PRE-ART INTERVIEW GUIDE
(FIELD-FORMATTED)

Thank you for agreeing to an interview today. My name is _______. We are conducting research about what helps and what prevents HIV-infected pregnant women from starting and adhering to ART. What helps will be called facilitators. What prevents or stops women will be called challenges or barriers.

Before we start, please write a response to the following basic demographic questions on the piece of paper provided to you. If needed, we can read the question to you and write your answer down on your behalf. If there is a question you do not want to answer, you can leave it blank. [Distribute appendix to complete.]

We hope to use the information shared today to create and/or design the ART readiness assessment tool and to develop or enhance programs to assist pregnant women in adhering to their medications or appointments. We envision the ART readiness assessment tool to consist of questions that a person answers before starting ART. We envision that the client's answers to the tool will guide the health care worker regarding ART initiation and how to help the client take the ART drugs and come to revisits.

Your ideas and experiences are very valuable. They will help us to understand how to improve adherence and clinic services. There are no right or wrong answers to our questions. We want to hear your ideas on this and learn about your experiences. Please be honest with us. We will also be speaking with other patients about their thoughts and experiences.

As we mentioned when you signed the consent form, we will be audio recording the discussion and taking notes throughout the discussion today. This is so we do not miss the important contributions that you have to make. Please do not be concerned about the audio recording. The discussion will remain confidential and will not be shared outside the research team.

The interview will last 1-2 hours. Do you have any questions before we begin?

Part A: Baseline Knowledge, Behavior, and Partner Involvement

<u>I will first ask you about your health and health care during your pregnancy:</u>

1. Can you describe your experience with antenatal care?
   Probes:
   - What do you think is a good number of antenatal care visits?
   - How many antenatal care visits do you plan to attend?

2. What are your plans for delivering the baby?
   Probes:
   - Do you plan to deliver at the health facility? Why or why not?

3. What are some things you know of that help prevent passing on HIV to your child?
   Probes:
   - Please tell us what you have heard about PMTCT (prevention of mother to child transmission of HIV).

<u>Now I would like to ask you about your own health</u>

4. As a pregnant woman with HIV, what are some things you do to stay healthy?
   Probe about:
   - diet, health care, ARVs

5. Please describe how your partner is involved in your health care.
   Probe:
   - Do you want more partner involvement or less? Please explain.

6. What are some challenges to accessing health care for you?
   Probes:
   - Please describe the impact of finances or costs on your use of health care.
   - *Similarly probe:* distance, transportation,(lack of) family support

7. The Zambian Ministry of Health recommends that all HIV-infected pregnant women start lifelong ARVs. What do you think about this idea?

8. Lifelong ARVs will mean taking medication every day. Have you ever taken medications on a daily basis for more than 2 weeks before? If so, can you describe that experience?
   Probes:
   - Give an example of a time when you missed a dose. Walk us through what happened.
   - What are some things you did to help you remember to take your medications?
   - How did others help you in taking your medication regularly?

Part B: ART Initiation

1. What are some reasons you think HIV-infected pregnant women feel ready to start taking ARVs?

2. What are some reasons you think HIV-infected pregnant women DO NOT feel ready to start taking ARVs?

3. Were you advised to take ARVs by your counselor? (If No, skip to number 5)

4. What were some reasons you have not started ARV medication?

5. What things do you think will help you to start ARV medication?
   Probe:
   - What have you seen are things that have helped others you know with HIV start to take ARVs?

6. What do you think will be some of the challenges to starting ARVs?
   Probe:
   - What have you seen are challenges to taking ARVs among others you know with HIV?

7. Barrier cards:

   On the table are cards that show reasons why people do not start ARVs when the health care worker recommends that they do start ARVs. I will read each one to you so that we can make sure that you understand what each one is. Please ask any questions. Please pick up the cards that represent the top 5 barriers to starting ARVs for you and put them in a separate pile. There are also a few blank cards for you to write on in case there are reasons that you do not see listed that you feel are important. [Give patient time to do this. Encourage them to limit their choices to 5 cards. Do not suggest any topics to be written onto the blank cards.]

   Now that you have selected these cards, I'd like to talk about them. Why does [read first card] cause problems for starting ART? Why does [read second card] cause problems for starting ARVs? [Ask the same question of each card until the pile, up to 5 cards, is finished.]

   Now that we have talked about them, please look at the cards again. Do you think there are other important reasons that you would like to add to your pile? Or would you like to put any of the original cards back? Why is this a barrier or no longer a barrier? [Give patient time to do this. Try to limit to 5 additional barriers.]

   If [point to one of the cards in the pile the patient chose and read it] were not true, what might still prevent you from starting ARVs? [Repeat question with 2-3 cards in the pile.]

Part C: ART Adherence
We have just finished talking about starting ARVs. Now we will shift to talking about the experience of taking ARVs regularly for a long period of time. Imagine that you have started ARVs and should take your ARV medication at the same time every day.

1. Why is it important that you take your medications every day at the same time?

2. What things do you think will help you to take your ARV medication regularly?
   Probe:
   - What have you seen are things that have helped others you know with HIV take ARVs?

3. What do you think will be some of the challenges to taking ARVs regularly?
   Probe:
   - What things might make you miss taking your medications? How often do these occur?
   - What have you seen are challenges to taking ARVs among others you know with HIV?

4. Barrier cards:

   On the table are cards that show reasons why people do not take their ART every day. Please pick up the cards that represent the top 5 barriers to taking ARVs regularly for you and put them in a separate pile. There are also a few blank cards for you to write on in case there are reasons that you do not see listed that you feel are important. [Give patient time to do this. Encourage them to limit their choices to 5 cards. Do not suggest any topics to be written onto the blank cards.]

   Now that you have selected these cards, I'd like to talk about them. Why does [read first card] cause problems for taking ARVs? Why does [read second card] cause problems for taking ARVs? [Ask the same question of each card until the pile, up to 5 cards, is finished.]

   Now that we have talked about them, please look at the cards again. Do you think there are other important reasons that you would like to add to your pile? Or would you like to put any of the original cards back? Why is this a barrier or no longer a barrier? [Give patient time to do this. Try to limit to 5 additional barriers.]

If [point to one of the cards in the pile the patient chose and read it] were not true, what might still prevent you from taking ARVs? [Repeat question with 2-3 cards in the pile.]

5. Which strategies would assist you in taking your ARVs regularly?
   Probes:
   - What strategies have you heard of for taking ARVs regularly ?
   - How do you think these strategies would work for you?

   (For the following, only cover the strategies they did not already mention)
   - What is your opinion on the following strategies for helping you take ARVs regularly:
     - Adherence counseling?
     - SMS reminders?
     - Follow up by peer educators?
     - Incentives?
     - Tracking CD4 and other test results?

6. If you could implement only one measure or strategy to help pregnant women remember to take their ARVs regularly, what would it be and why?

Conclusion

Before we finish, is there anything else you would like to say about the topics we discussed today?

Thank you so much for taking the time to help with this. Your suggestions and comments are very valuable and will be used to improve efforts to support ART initiation and adherence among pregnant women. If you have any questions after today or if you would like to learn more about the results of the study, you can contact Dr. Mubiana-Mbewe at +260 1 293 661.

Participant ID number: ☐☐☐☐☐

Date: ☐☐/☐☐/☐☐☐☐

Appendix A: Basic demographic data on interview participants (pre-ART)

Instructions: Kindly circle your answer choice or write responses in the space provided. If there is a question you do not want to answer, you can leave it blank. Do not write your name or surname on this form. Thank you.

1. What tribe do you belong to? ___________________

2. In which province is this tribe?
   a. Central
   b. Copperbelt
   c. Eastern
   d. Luapula
   e. Lusaka
   f. Muchinga
   g. Northern
   h. Northwestern
   i. Southern
   j. Western

3. How old are you? ________ years old

4. What is your highest level of education that you have completed?
   a. None
   b. Primary
   c. Secondary
   d. Tertiary

5. What is your marital status?
   a. Single or never married
   b. Married or co-habiting
   c. Divorced or separated
   d. Widowed

6. What is your primary occupation?
   a. Not employed or keeps household
   b. Occasionally employed, such as day-worker
   c. Informally employed
   d. Business woman or self-employed
   e. Formally employed in someone's household
   f. Formally employed in organization, company or institution

7. What is your monthly income?
   a. Less than ZMW 250.00
   b. ZMW 250.00 to ZMW 499.99
   c. ZMW 500.00 to ZMW 999.99
   d. ZMW 1000.00 to ZMW 1999.99
   e. ZMW 2000.00 to ZMW 5000.00
   f. More than ZMW 5000.00

Participant ID number: ☐☐☐☐☐

Date: ☐☐/☐☐/☐☐☐☐

8. How many times have you given birth to a baby at 7 months gestation or over? ________

9. How many living children do you have right now? ________ living children

10. How long have you known that you have the HIV infection? ________ months (if less than 1 month, then write here: ________ weeks)

11. When do you plan to start ART?
    a. In less than 1 month from today
    b. In 1 month to 3 months
    c. In 3 months to 6 months
    d. In 6 months to 12 months
    e. After 12 months

12. Which clinic will you attend to receive your HIV care? ___________________ health facility

13. How far away do you live from that clinic? ________ minutes by foot to reach the clinic from home
