# Supplementary material for: Identifying barriers to ART initiation and adherence: An exploratory qualitative study on PMTCT in Zambia
Source: PLoS One. 2022 Jan 13;17(1):e0262392. doi: 10.1371/journal.pone.0262392 (PMC8757984; doi:10.1371/journal.pone.0262392)
Supplement: S2 File — (PDF) [file pone.0262392.s003.pdf]

Participant ID number: ☐☐☐☐☐

Date: ☐☐/☐☐/☐☐☐☐

ART Readiness among Pregnant Women (Phase 1),
Interview Guide, Version 1.2, dated 13 April 2014

WOMEN ON ART INTERVIEW GUIDE
(FIELD-FORMATTED)

Thank you for agreeing to an interview today. My name is _______. We are conducting research about what helps and what prevents HIV-infected pregnant women from starting and adhering to ARV medication. What helps will be called facilitators. What prevents or stops women will be called challenges or barriers.

Before we start, please write a response to the following basic demographic questions on the piece of paper provided to you. If needed, we can read the question to you and write your answer down on your behalf. If there is a question you do not want to answer, you can leave it blank. [Distribute appendix to complete.]

We hope to use the information shared today to create and/or design the ARV readiness assessment tool and to develop or enhance programs to assist pregnant women in adhering to their medications or appointments. We envision the ARV readiness assessment tool to consist of questions that a person answers before starting ARVs. We envision that the client's answers to the tool will guide the health care worker regarding ARV initiation and how to help the client take the ARV drugs and come to revisits.

Your ideas and experiences are very valuable. They will help us to understand how to improve adherence and clinic services. There are no right or wrong answers to our questions. We want to hear your ideas on this and learn about your experiences. Please be honest with us. We will also be speaking with other patients about their thoughts and experiences.

As we mentioned when you signed the consent form, we will be audio recording the discussion and taking notes throughout the discussion today. This is so we do not miss the important contributions that you have to make. Please do not be concerned about the audio recording. The discussion will remain confidential and will not be shared outside the research team.

The interview will last 1-2 hours. Do you have any questions before we begin?

Part A: Baseline Knowledge, Behavior, and Partner Involvement
<u>I will first ask you about your health and health care during your pregnancy:</u>

1. Can you describe your experience with antenatal care?
   Probes:
   - What do you think is a good number of antenatal care visits?
   - How many antenatal care visits do you plan to attend?

2. What are your plans for delivering the baby?
   Probes:
   - Do you plan to deliver at the health facility? Why or why not?

3. What are some things you know of that help prevent passing on HIV to your child?
   Probes:
   - Please tell us what you have heard about PMTCT (prevention of mother to child transmission of HIV).

<u>Now I would like to ask you about your own health</u>

4. As a pregnant woman with HIV, what are some things you do to stay healthy?
   Probe about:
   - diet, health care, ARVs

5. Please describe how your partner is involved in your health care.
   Probe:
   - Do you want more partner involvement or less? Please explain.

6. What are some challenges to accessing health care for you?
   Probes:
   - Please describe the impact of finances or costs on your use of health care.
   - *Similarly probe:* distance, transportation,(lack of) family support

7. The Zambian Ministry of Health recommends that all HIV-infected pregnant women start lifelong ARVs. What do you think about this idea?

Part B: ART Initiation
<u>Now I want you think back to when you first started taking ARVs.</u>

1. What was it like for you to start ARVs?
   Probe:
   - When did you start ARVs? How long have you been on ARVs?

2. How did your starting date for ARVs compare to what the healthcare worker recommended for you?
   Probes:
   - If there was a delay between the recommended start date and when you actually started, what were some of the reasons for this delay?

3. What were the things that helped you to first start ARV medication?

4. What were some of the challenges you faced when first starting ARVs?

5. Barrier cards:

   On the table are cards that show reasons why people do not start ARVs when the health care worker recommends that they do start ARVs. I will read each one to you so that we can make sure that you understand what each one is. Please ask any questions. Please pick up the cards that represent the top 5 barriers to starting ARVs for you and put them in a separate pile. There are also a few blank cards for you to write on in case there are reasons that you do not see listed that you feel are important. [Give patient time to do this. Encourage them to limit their choices to 5 cards. Do not suggest any topics to be written onto the blank cards.]

   Now that you have selected these cards, I'd like to talk about them. Why does [read first card] cause problems for starting ARVs? Why does [read second card] cause problems for starting ARVs? [Ask the same question of each card until the pile, up to 5 cards, is finished.]

   Now that we have talked about them, please look at the cards again. Do you think there are other important reasons that you would like to add to your pile? Or would you like to put any of the original cards back? Why is this a barrier or no longer a barrier? [Give patient time to do this. Try to limit to 5 additional barriers.]

If [point to one of the cards in the pile the patient chose and read it] were not true, what might still prevent you from starting ARVs? [Repeat question with 2-3 cards in the pile.]

Part C: ART Adherence

We have just finished talking about starting ARVs. Now we will shift to talking about the experience of taking ARVs regularly for a long period of time.

1. Why is it important that you take your ARV medications every day at the same time?

2. Since you started taking ARVs, when did you find it most difficult to take your medications every day? Tell us about this time.

3. At this point, what are some challenges you continue to face in taking ARVs regularly?
   Probes:
   - What are some things that lead you to miss one or more doses? How often does this occur?
   - Can you give us an example of a time when you missed a dose? Tell us what happened.

   - What are some things that lead you to miss pharmacy refill or clinic appointments? How often does this occur?
   - Can you give us an example of a time when you missed a pharmacy or clinic appointment? Tell us what happened.

4. Barrier Cards

   On the table are cards that show reasons why people do not take their ARVs every day. Please pick up the cards that represent the top 5 barriers to taking ARVs for you and put them in a separate pile. There are also a few blank cards for you to write on in case there are reasons that you do not see listed that you feel are important. [Give patient time to do this. Encourage them to limit their choices to 5 cards. Do not suggest any topics to be written onto the blank cards.]

Now that you have selected these cards, I'd like to talk about them. Why does [read first card] cause problems for taking ARVs? Why does [read second card] cause problems for taking ARVs? [Ask the same question of each card until the pile, up to 5 cards, is finished.]

Now that we have talked about them, please look at the cards again. Do you think there are other important reasons that you would like to add to your pile? Or would you like put any of the original cards back? Why is this a barrier or no longer a barrier? [Give patient time to do this. Try to limit to 5 additional barriers.]

If [point to one of the cards in the pile the patient chose and read it] were not true, what might still prevent you from taking ARVs? [Repeat question with 2-3 cards in the pile.]

<u>Now that we have talked about some challenges you face, lets discuss what things help you to take your ARVs every day.</u>

5. At this point, what are some things that help you to take your ARV medication regularly?
   Probes:
   - What do you do to remember to take your medications? How does this help you to take them at the same time every day?
   - How do others help you in taking your medication regularly?
   - How do you know if you took your ARVs yesterday?

6. Adherence counseling is one strategy that can help someone take their medications on time. Adherence counseling is when a patient meets one-on-one with a peer educator before the patient gets ARV medications or sees a nurse or doctor. Do you remember this ever happening? *(If no, skip to 6h)*
   a. What topics were covered in adherence counseling?

   b. What else is done in adherence counseling?

   c. What do you like about adherence counseling? Why?

   d. What do you not like about adherence counseling? Why not?

e. How would you describe your relationship with the peer educator(s) who conducted adherence counseling?
    i. Did they respect you?
    ii. Did they listen to you?
    iii. Did they judge you based on what you say during adherence counseling? Does this affect what you share with them?
    iv. During the adherence counseling sessions, who talked more: you or the peer educator?
    v. Did you ever ask questions during these sessions? If so, what did you ask about? If not, why not?

f. How have adherence counseling sessions changed the way you take your ARVs?

g. If you could, what would you change about adherence counseling? Why?

(Ask if they did NOT receive adherence counseling)

h. If you did not participate in adherence counseling, would you still be interested in doing so? Why or why not?

7. Which strategies assist you in taking your ARVs regularly?
   Probes:
   - What strategies have you heard of for taking ARVs regularly?
   - How do you think these strategies would work for you?

   (For the following, only cover the strategies they did not already mention)
   - What is your opinion on the following strategies for helping you take ARVs regularly:
     - SMS reminders?
     - Follow up by peer educators?
     - Incentives?
     - Tracking CD4 and other test results?
   - Which strategy do you think would work best for you? Why?

8. If you could implement only one measure or strategy to help pregnant women remember to take their ARVs regularly, what would it be and why?

Conclusion

Before we finish, is there anything else you would like to say about the topics we discussed today?

Thank you so much for taking the time to help with this. Your suggestions and comments are very valuable and will be used to improve efforts to support ARV initiation and adherence among pregnant women. If you have any questions after today or if you would like to learn more about the results of the study, you can contact Dr. Mubiana-Mbewe at +260 1 293 661.

Flesch-Kincaid Grade Level: 5.5

Participant ID number: ☐☐☐☐☐

Date: ☐☐/☐☐/☐☐☐☐

Appendix A: Basic demographic data on interview participants (post-ART)

Instructions: Kindly circle your answer choice or write responses in the space provided. If there is a question you do not want to answer, you can leave it blank. Do not write your name or surname on this form. Thank you.

1. What tribe do you belong to? ___________________

2. In which province is this tribe?
    a. Central
    b. Copperbelt
    c. Eastern
    d. Luapula
    e. Lusaka
    f. Muchinga
    g. Northern
    h. Northwestern
    i. Southern
    j. Western

3. How old are you? ________ years old

4. What is your highest level of education that you have completed?
    a. None
    b. Primary
    c. Secondary
    d. Tertiary

5. What is your marital status?
    a. Single or never married
    b. Married or co-habiting
    c. Divorced or separated
    d. Widowed

6. What is your primary occupation?
    a. Not employed or keeps household
    b. Occasionally employed, such as day-worker
    c. Informally employed
    d. Business woman or self-employed
    e. Formally employed in someone's household
    f. Formally employed in organization, company or institution

7. What is your monthly income?
    a. Less than ZMW 250.00
    b. ZMW 250.00 to ZMW 499.99
    c. ZMW 500.00 to ZMW 999.99
    d. ZMW 1000.00 to ZMW 1999.99
    e. ZMW 2000.00 to ZMW 5000.00
    f. More than ZMW 5000.00

8. How many times have you given birth to a baby at 7 months gestation or over? ________

9. How many living children do you have right now? ________ living children

10. How long have you known that you have the HIV infection? ________ months (if less than 1 month, then write here: ________ weeks)

11. How long have you been taking ART? ________ months (if less than 1 month, then write here: ________ weeks)

12. Are you on 'first line' or 'second line' medications?
    a. First line ART medications
    b. Second line ART medications
    c. I don't know

13. When do you plan to start ART?
    a. In less than 1 month from today
    b. In 1 month to 3 months
    c. In 3 months to 6 months
    d. In 6 months to 12 months
    e. After 12 months

14. Which clinic will you attend to receive your HIV care? ___________________ health facility

15. How far away do you live from that clinic? ________ minutes by foot to reach the clinic from home
