# Supplementary material for: Identifying barriers to ART initiation and adherence: An exploratory qualitative study on PMTCT in Zambia
Source: PLoS One. 2022 Jan 13;17(1):e0262392. doi: 10.1371/journal.pone.0262392 (PMC8757984; doi:10.1371/journal.pone.0262392)
Supplement: S3 File — (PDF) [file pone.0262392.s004.pdf]

**ART Readiness among Pregnant Women (Phase 1), Cards for Interview,**

**Version 1.0, dated 8 November 2013**

| | | | |
|---|---|---|---|
| My partner does not know that I'm HIV positive. | I am taking other treatment that may cure me from a traditional healer. | My community says bad things about people who are HIV-infected or are taking ART. | The clinic is too far away from my home. |
| My family does not support me to take ART. | I cannot get time off from work to get ART refills from the clinic. | My church does not support me to take ART. | My partner does not support me to take ART. |
| I have not told anyone that I'm HIV positive. | The queue for medications at the clinic is too long, and I cannot wait for so long. | I don't want anyone to know that I'm HIV positive. | I run out of ART medication. |
| I feel healthy. | I do not know when I am supposed to take my ART medication. | Taking ART has side effects that make me not want to take them. | I feel too sick to go to clinic or take my ART medication. |

| | | | |
|---|---|---|---|
| **I think other treatments work better than ART medication.** | **I believe that I have been cursed, and so ART medications will not help.** | **I am not paying for the ART medication so it is not important that I take each pill.** | **I cannot pay for transport to get to the clinic.** |
| **There is no one whom I can ask questions when I have problems with my ART medication.** | **I believe I will die anyway so there is no reason to take medication every day.** | **I feel sad and depressed.** | **I travel a lot.** |
| **I just forget to take my ART medication.** | **I am too busy to take my ART medication.** | | |
